# Telehealth Demand Trends During the COVID-19 Pandemic in the Top 50 Most Affected Countries: Infodemiological Evaluation

**DOI:** 10.2196/24445

**Published:** 2021-02-19

**Authors:** Mark Yu Zheng Wong, Dinesh Visva Gunasekeran, Simon Nusinovici, Charumathi Sabanayagam, Khung Keong Yeo, Ching-Yu Cheng, Yih-Chung Tham

**Affiliations:** 1 School of Clinical Medicine University of Cambridge Cambridge United Kingdom; 2 Singapore Eye Research Institute Singapore National Eye Centre Singapore Singapore; 3 Department of Ophthalmology Yong Loo Lin School of Medicine National University of Singapore Singapore Singapore; 4 Duke-NUS Medical School National University of Singapore Singapore Singapore; 5 National Heart Centre Singapore Singapore Singapore

**Keywords:** COVID-19, infodemiology, telehealth, telemedicine, internet

## Abstract

**Background:**

The COVID-19 pandemic has led to urgent calls for the adoption of telehealth solutions. However, public interest and demand for telehealth during the pandemic remain unknown.

**Objective:**

We used an infodemiological approach to estimate the worldwide demand for telehealth services during COVID-19, focusing on the 50 most affected countries and comparing the demand for such services with the level of information and communications technology (ICT) infrastructure available.

**Methods:**

We used Google Trends, the Baidu Index (China), and Yandex Keyword Statistics (Russia) to extract data on worldwide and individual countries’ telehealth-related internet searches from January 1 to July 7, 2020, presented as relative search volumes (RSV; range 0-100). Daily COVID-19 cases and deaths were retrieved from the World Health Organization. Individual countries’ ICT infrastructure profiles were retrieved from the World Economic Forum Report.

**Results:**

Across the 50 countries, the mean RSV was 18.5 (SD 23.2), and the mean ICT index was 62.1 (SD 15.0). An overall spike in worldwide telehealth-related RSVs was observed from March 11, 2020 (RSV peaked to 76.0), which then tailed off in June-July 2020 (mean RSV for the period was 25.8), but remained higher than pre-March RSVs (mean 7.29). By country, 42 (84%) manifested increased RSVs over the evaluation period, with the highest observed in Canada (RSV=100) and the United States (RSV=96). When evaluating associations between RSV and the ICT index, both the United States and Canada demonstrated high RSVs and ICT scores (≥70.3). In contrast, European countries had relatively lower RSVs (range 3.4-19.5) despite high ICT index scores (mean 70.3). Several Latin American (Brazil, Chile, Colombia) and South Asian (India, Bangladesh, Pakistan) countries demonstrated relatively higher RSVs (range 13.8-73.3) but low ICT index scores (mean 44.6), indicating that the telehealth demand outstrips the current ICT infrastructure.

**Conclusions:**

There is generally increased interest and demand for telehealth services across the 50 countries most affected by COVID-19, highlighting the need to scale up telehealth capabilities, during and beyond the pandemic.

## Introduction

COVID-19 was formally declared a pandemic by the World Health Organization (WHO) on March 11, 2020. As of September 7, the WHO has reported over 27 million cases, with a cumulative mortality rate of 3.26% [1]. In the context of infectious disease outbreaks such as the current COVID-19 pandemic, concerns regarding the overloading of health care facilties, coupled with the need to minimize patient and health care provider exposure in hospital care settings have led to calls for a shift from the traditional patient-physician face-to-face physical consultations to telehealth-based remote clinical services [2-6]. However, the magnitude of this major shift in health care management has yet to be evaluated. Public interest in and potential demand for telehealth services are relatively unknown [7,8]. This information gap poses challenges for health care providers to redesign strategies, institute new policies, and restructure manpower and infrastructure to address a potential “new wave” of clinical needs.

Infodemiology is a rapidly growing field of methodology in health informatics, which study trends in online search behavior and internet activity [9,10]. These methods provide new insights on population behavior and health-related phenomena, particularly during infectious disease outbreaks [11-13]. Google Trends (GT) and the Baidu Index are examples of infodemiological tools that researchers have used to analyze temporal and geographical trends in relative search volume (RSV) on the internet, with GT being the most prolific among published reports [14-16]. These tools have the additional advantage of providing real-time data, reflecting the immediate changes in population behavior in response to real-world events [9,10]. In the current climate of the COVID-19 pandemic, these tools have recently been used to investigate the overall public interest in COVID-19 [17], public fear of COVID-19 symptoms [18], and changes in behavioral attitudes toward activies such as social distancing and hand washing [19].

A recent paper by Hong et al [2] utilized a similar infodemiological approach to describe the increase in telehealth-related search volumes in the United States, up to March 2020. Building upon this work, we further broadened our current investigation toward a global perspective and extended the evaluation period beyond the initial wave of COVID-19 to include postlockdown periods and the reopening of countries’ economy, society, and health care systems.

To provide a broader understanding of the current global interest and demand for telehealth, we used an infodemiological approach to investigate internet RSV as a proxy for public interest and demand for telehealth services in the 50 countries most affected by COVID-19. We described trends in telehealth-related RSVs across these countries over a 6-month period, spanning from the start of the pandemic to each country’s lockdowns and their subsequent reopening. Finally, we compared the demand for telehealth with the level of information and communications technology (ICT) infrastructure for each country. These findings may provide valuable information for policy makers and health care providers to better cater to the new demands for telehealth services during COVID-19, and into the post–COVID-19 new normal.

## Methods

### Retrieving COVID-19 Key Dates and Confirmed Case Numbers

Real-world data on daily confirmed COVID-19 cases and deaths were retrieved on July 9, 2020, from the WHO’s COVID-19 dashboard from January 1, 2020, until July 7, 2020 [1]. Worldwide data, as well as individual country-level data for the 50 countries with the highest cumulative confirmed COVID-19 case numbers (as of July 7, 2020), were also retrieved. Key dates of the COVID-19 pandemic were retrieved from the WHO’s COVID-19 timeline and news reports of regional COVID-19–related events [20,21].

### Retrieving Data From GT and Other Country-Specific Search Query Databases

GT provides data on volumes and patterns in online search behaviors of internet users [15]. It tracks keyword search queries that users enter into the Google search engine and presents information on the search query according to the specified time period and geographical location [22]. The search volume results are normalized and presented as an RSV index, wherein each data point is divided by the total searches performed in a specified geography within a given time range to provide relative comparisons [22]. The resulting output ranges from 0 to 100, with 100 indicating the maximum search interest in the selected time period and location. To comprehensively capture trends in online search behavior and infectious burden over an extended period, daily worldwide and country-specific GT data were retrieved over the first 6 months of the outbreak, from January 1 to July 7, 2020.

In addition to GT, the Baidu Index and the Yandex Wordstat Keyword Statistics Service were used to retrieve data on search queries in China and Russia, respectively. Baidu and Yandex are the predominant search engines used in China and Russia, respectively [14,23]. To facilitate direct visualization and comparisons with the RSV index obtained from GT, data from the Baidu Index and Yandex were similarly scaled to range from 0 to 100 [18]. In this work, total-RSV denotes the cumulative RSVs over the entire evaluation period, while average RSV represents an RSV value that was averaged over a specified period (eg, pre– or post–COVID-19 period; further descriptions are provided below).

### Keyword Selection

For the GT analysis, we followed the detailed methodology described by Mavragani et al [10] for our keyword selection. First, different permutations of search terms and topics related to “telehealth” and “telemedicine” were searched to understand overarching trends in worldwide interest and to optimize keyword search combinations. We then conducted worldwide and country-specific GT searches using a baseline combination of English, Spanish, Russian, and French translations (chosen from the list of most commonly spoken languages worldwide) [24], in addition to translations in the native or official language of that particular country. Mandarin Chinese is the second most spoken language in the world [24] but was not included in the baseline search combination since the majority of native Mandarin Chinese speakers reside in China and do not use Google as their main internet search tool. For the Baidu and Yandex search indexes, a combination of keywords in Mandarin Chinese (both traditional and simplified) and Russian, respectively, were used. The detailed keyword search strategy can be found in Table S1 in Multimedia Appendix 1.

### Retrieval of Additional Telehealth-Related National Indicators

Data regarding the key dates of major public health responses such as lockdowns for each country were obtained from internet sources and news reports [25-27]. In addition, ICT data from the ICT adoption pillar of the Global Competitiveness Index 4.0 framework were obtained from the World Economic Forum Global Competitiveness Report 2019 and used to compare ICT level across countries [28]. The extracted ICT adoption index (ICT index) scores range from 0 to 100 (highest), wherein a higher score represents greater levels of networked infrastructure and higher regional usage and access to such infrastructure. Additional country-specific indices including GDP (gross domestic product) per capita ($US), literacy rates, and World Bank regional income groups were obtained from the World Bank DataBank [29].

As a proxy measure of the existing telehealth capacity of the respective countries, the Crunchbase (CB) [30] and CB Insights (CBI) [31] business analytic platforms were used to search for the prevalence of prominent telehealth providers within each of the 50 countries, and was used to define a CB/CBI score.

### Analysis

First, to provide a more accurate observation of underlying trends and eliminate short-term fluctuations in data, the time trends for telehealth-related RSVs were smoothened by 7-day rolling intervals [32]. For countries with lower total-RSVs (<5), 14-day interval smoothing was used instead, as these countries are more susceptible to daily fluctuations (thus more “noise” in the trend data). Having a longer smoothing interval helps to minimize errors in estimations caused by these fluctuations over a short period. The RSVs were then plotted against daily COVID-19 confirmed cases and deaths (two separate Y axes), both worldwide and for each of the 50 countries.

Mean (SD) and median (IQR) values were used to provide summary statistics for key country parameters including total-RSVs, COVID-19 cases and deaths, and ICT indexes. The Pearson and Spearman correlation tests (when applicable) were used to further investigate associations between key parameters. The kurtosis/skewness and Shapiro-Wilk tests were used to determine normality of data distribution. *P* values of <.05 were considered as statistically significant.

The lockdown periods for each country were incorporated along with two key dates for reference—January 23, 2020, when China first imposed a lockdown in Hubei Province, and March 11, 2020, when the WHO declared COVID-19 a global pandemic. When evaluating changes in average RSV levels in the pre– and post–COVID-19 periods (ie, RSVs averaged over the period before and after COVID-19, respectively), we defined these periods based on the landmark date of March 11, 2020 (the WHO’s COVID-19 pandemic declaration), except for China, where the pre–COVID-19 period was defined as before January 23, 2020. The ratios of the average pre– and post–COVID-19 RSV levels for each country were then calculated.

Lastly, bubble plots were used to illustrate the relationships between the total-RSVs of individual countries and various telehealth-related national indicators (GDP per capita [$US], literacy rates, the ICT index, and the CB/CBI score). Countries were grouped and color-coded according to World Bank regional classifications. All analyses and visualizations were conducted using Python (Python Software Foundation, version 3.7.4).

## Results

**Figure 1:**
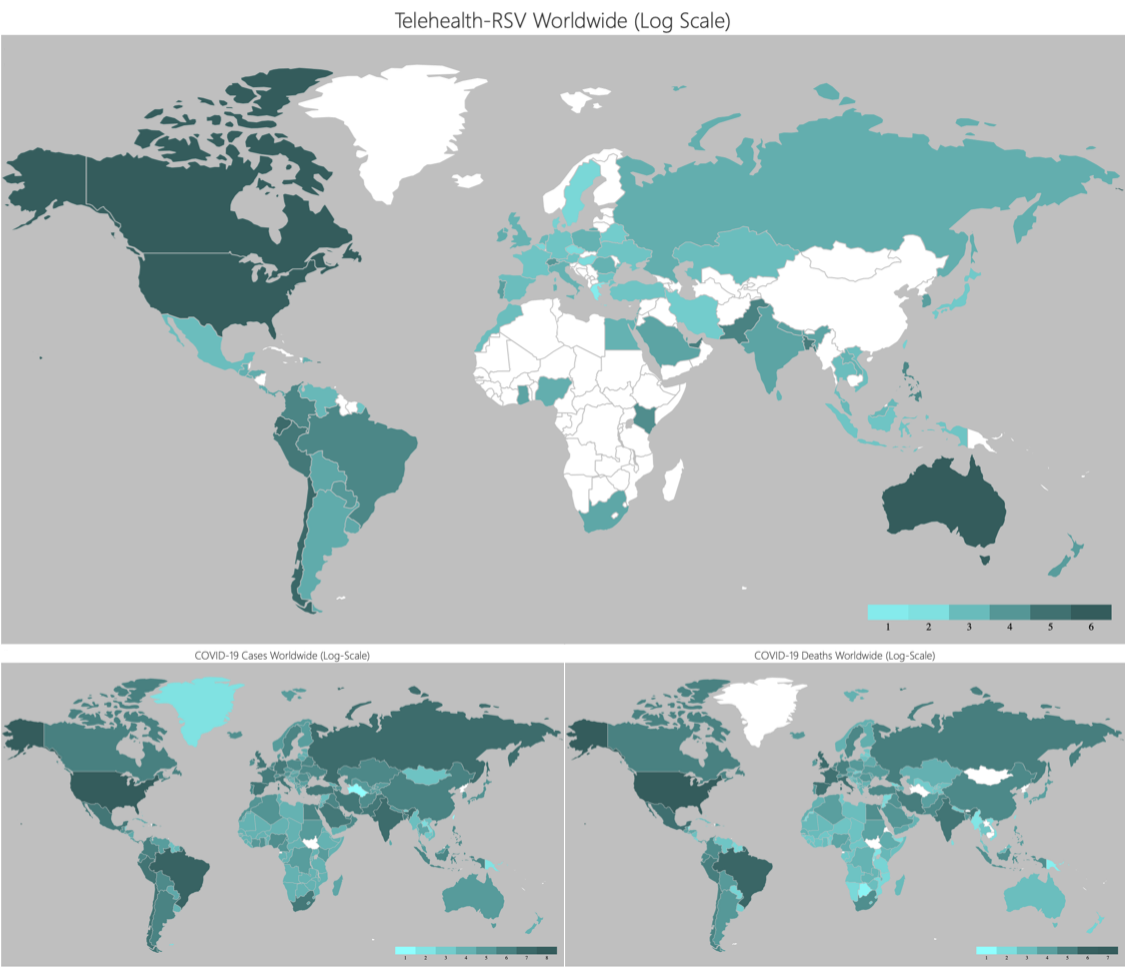
Global choropleth map comparing telehealth-related relative search volumes (RSVs) (top), real-world COVID-19 confirmed cases (bottom left), and real-world COVID-19 confirmed deaths (bottom right).

**Figure 2:**
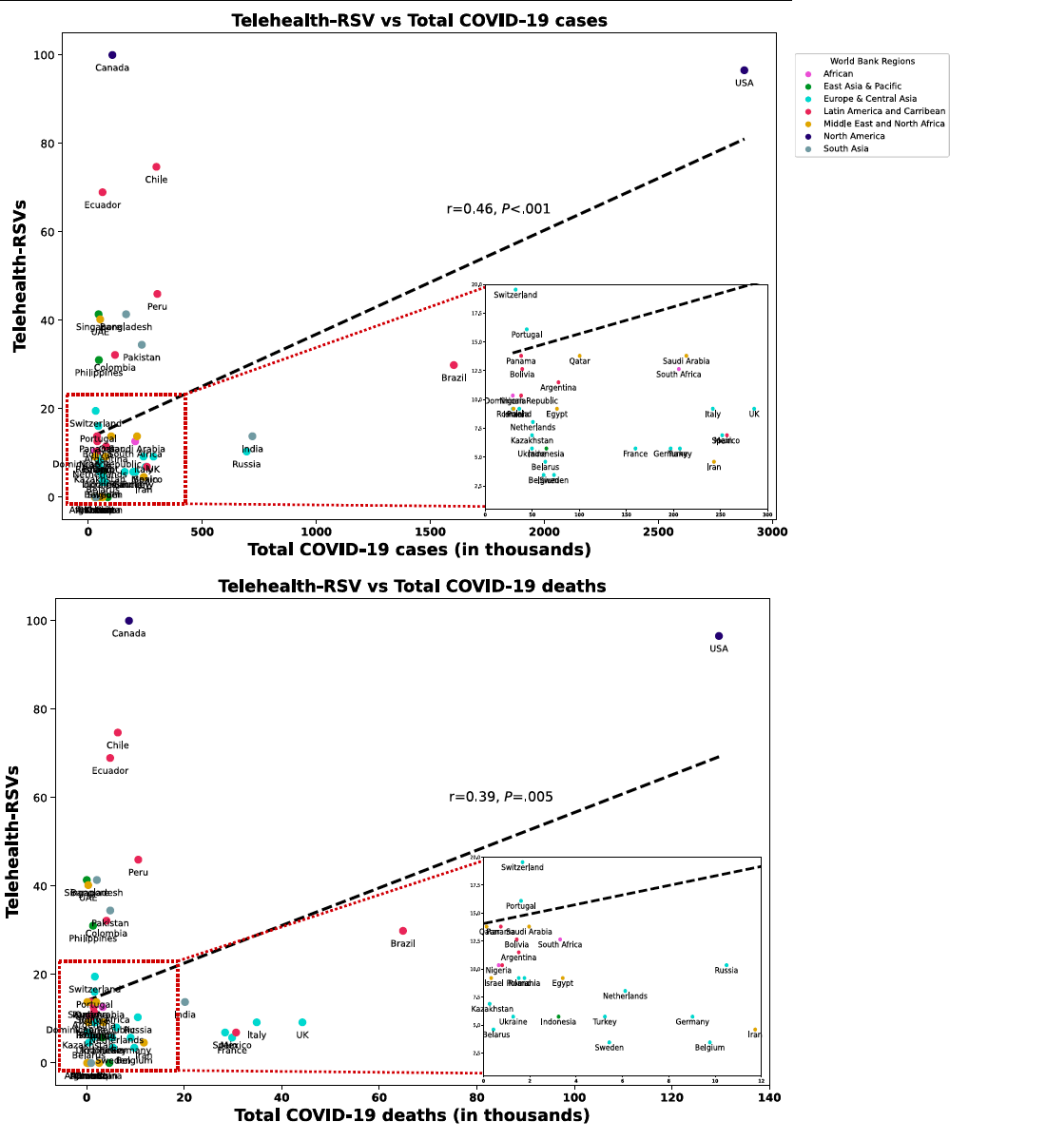
Correlation between total telehealth-related relative search volumes (RSVs) against total COVID-19 cases (left) and deaths (right) per country.

Characteristics of the 50 countries, including the number of COVID-19 cases and deaths, telehealth-related RSVs, and ICT index values, are presented in Table 1. Across the 50 countries, the mean total-RSV was 18.5 (SD 23.2; median 9.20, IQR 5.75–18.68), and the mean ICT index score was 62.1 (SD 15.0; median 64.5, IQR 51.2–72.5). Figure 1 (top) shows a geographic choropleth map of the telehealth-related GT RSVs. North American countries had the highest total-RSVs (RSV=100 in Canada and RSV=96.6 in the United States). Within Europe, Switzerland (RSV=19.5) and Portugal (RSV=16.1) had the highest total-RSVs. For Latin America and the Carribean region, Chile (RSV=74.7) and Ecuador (RSV=69.0) had the highest total-RSVs. Likewise, the United Arab Emirates (RSV=40.2) scored the highest for the Middle East, South Africa (RSV=12.6) for sub-Saharan Africa, Bangladesh (RSV=41.4) for South Asia, and Singapore (RSV=41.4) for East Asia. Similarly, Figure 1 (bottom row) demonstrates geographic choropleth maps by the number of COVID-19 confirmed cases and deaths, respectively. Overall, among the evaluated countries, there were fair correlations between total-RSVs and COVID-19 cases (Pearson *r*=0.46, *P*<.001; Spearman ρ=0.29, *P*=.04) and deaths (*r*=0.39, *P*=.005; ρ=0.17, *P*=.25) (Figure 2).

**Table 1:** Key COVID-19 and telehealth-related parameters for the top 50 countries most affected by the pandemic.

Rank	Country	COVID-19 cases^a^, n	COVID-19 deaths^a^, n	Total-RSVs^b^	ICT^c^ adoption index
1	United States	2,877,238	129,643	96.6	74.35
2	Brazil	1,603,055	64,867	29.9	58.06
3	India	719,665	20,160	13.8	32.11
4	Russia	694,230	10,494	10.3^d^	77.03
5	Peru	302,718	10,589	46.0	45.70
6	Chile	298,557	6384	74.7	63.13
7	United Kingdom	285,772	44,236	9.2	72.99
8	Mexico	256,848	30,639	6.9	55.03
9	Spain	251,789	28,388	6.9	78.21
10	Iran	243,051	11,731	4.6	50.85
11	Italy	241,819	34,869	9.2	64.49
12	Pakistan	234,509	4839	34.5	25.21
13	Saudi Arabia	213,716	1968	13.8	69.30
14	Turkey	206,844	5241	5.7	57.82
15	South Africa	205,721	3310	12.6	49.67
16	Germany	196,944	9024	5.7	69.98
17	Bangladesh	165,618	2096	41.4	39.14
18	France	159,568	29,831	5.7	73.66
19	Colombia	117,110	4064	32.2	49.89
20	Canada	105,536	8684	100.0	70.29
21	Qatar	100,345	133	13.8	83.78
22	China	85,345	4648	52.9^e^	78.49
23	Argentina	77,815	1523	11.5	57.99
24	Egypt	76,222	3422	9.2	40.57
25	Sweden	73,061	5433	3.42	87.78
26	Indonesia	64,958	3241	5.7	55.37
27	Belarus	63,804	429	4.6	—^f^
28	Ecuador	62,380	4821	69.0	47.62
29	Iraq	62,275	2567	0	—
30	Belgium	62,058	9774	3.4	67.02
31	United Arab Emirates	52,068	324	40.2	91.87
32	Netherlands	50,602	6119	8.0	76.29
33	Kuwait	50,644	373	0	69.57
34	Ukraine	49,607	1283	5.7	51.85
35	Kazakhstan	49,683	264	6.9	67.99
36	Oman	47,735	218	0	58.11
37	Philippines	46,333	1303	31.0	49.69
38	Singapore	44,983	26	41.4	87.11
39	Portugal	44,129	1620	16.1	71.24
40	Panama	38,149	747	13.8	50.06
41	Bolivia	39,297	1434	12.6	51.45
42	Dominican Republic	38,128	804	10.3	51.79
43	Poland	36,155	1521	9.2	65.41
44	Afghanistan	33,384	920	0	—
45	Switzerland	32,230	1685	19.5	78.58
46	Israel	30,055	331	9.2	67.56
47	Bahrain	29,821	98	0	67.19
48	Nigeria	29,286	654	10.3	33.39
49	Armenia	29,285	503	0	62.02
50	Romania	29,223	1768	9.2	72.01

^a^COVID-19 case and death numbers as of July 7, 2020.

^b^RSV: relative search volume. Total-RSVs were calculated over the evaluation period from January 1 to July 7, 2020.

^c^ICT: information and communications technology.

^d^For Russia, the RSV value reflects search volumes as measured by GT only. Mean search volumes based on Yandex was 44.3 but was not listed in the table.

^e^For China, the RSV value reflects the mean RSV as measured by the Baidu Index. GT was not applicable for China.

^f^Not available.

**Figure 3:**
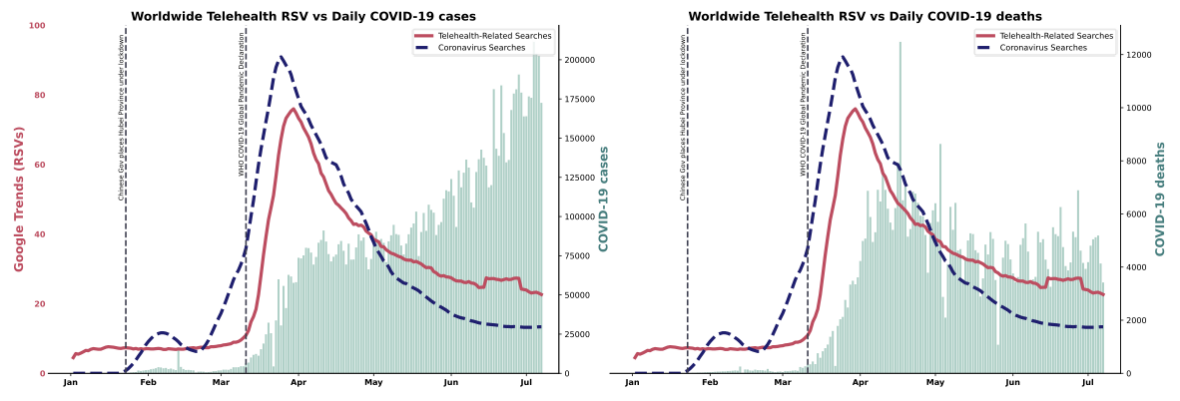
Worldwide time trends for telehealth-related relative search volumes (RSVs) (red) against daily COVID-19 cases (left) and deaths (right). The x axis represents time in individual days from January 1 to July 7, 2020. The left and right y axes represent Google Trends RSVs and COVID-19 cases or deaths, respectively. Blue and red trendlines represent “coronavirus” and telehealth-RSVs, respectively. Teal vertical bars represent daily COVID-10 cases or deaths. Black vertical lines represent two key dates: the start of the Hubei Province lockdown (January 23, 2020) and the declaration of COVID-19 as a pandemic by the World Health Organization (WHO) on March 11, 2020.

Figure 3 depicts the overall worldwide trends in telehealth-related RSVs during the study period, on a backdrop of accumulated COVID-19 cases and deaths. A surge in RSV levels can be observed from March 11, 2020, the date when the WHO officially declared the COVID-19 outbreak a pandemic, and culminates with an observed peak (RSV=76.0) in telehealth-RSV levels on March 24, 2020, with more than 10 times the pre–COVID-19 levels (mean 7.29). RSV levels then tailed off in June-July 2020 (mean 25.8) but still remained higher than pre–COVID-19 levels. For comparison, another curve representing RSVs for a search of “coronavirus” (in blue) was plotted alongside the telehealth-RSV curve (in red). The coronavirus-related RSVs demonstrated a similar trend—a sharp spike near March 11 and peaking on March 18.

Country-specific telehealth-RSV levels during the study period were similarly plotted in Multimedia Appendix 2. In total, 42 (84%) of the evaluated countries manifested spikes in telehealth-RSV levels over the evaluation period. When comparing the average pre– and post–COVID-19 RSV levels across these 42 countries, RSV levels increased by an average of 4.07 (SD 3.23) times post COVID-19 (Table S2, Multimedia Appendix 1). The remaining 8 countries were from Central Asia (Armenia) and the Middle East (Iran, Belarus, Iraq, Kuwait, Oman, Afghanistan, Bahrain), and had either no RSVs or no observable increased trends.

Of the 42 countries with increases in RSVs, the United States and Canada had the highest total-RSVs, showing sharp increases in telehealth-RSV levels during early March, peaking in mid to late March, before decreasing and eventually plateauing to levels that were still higher than pre–COVID-19 levels (Multimedia Appendix 2). Compared to pre–COVID-19 levels, the average RSV levels increased by 5.83 times in the United States and by 2.69 times in Canada (Table S2, Multimedia Appendix 1). The remaining 40 countries also displayed increases in RSV levels over the evaluation period, albeit with less prominent spikes compared to the United States and Canada. These countries often approached peak RSVs more gradually over a longer period of months. These countries can be further subdivided into two categories based on the magnitude of RSV increases (Table S2, Multimedia Appendix 1). The first group observed large increases in average RSV levels (≥2.5 times increase compared to pre–COVID-19 levels; n=24). This group consisted largely of countries from Latin America (Brazil, Peru, Chile, Colombia, Argentina, Ecuador), South Asia (India, Bangladesh, Pakistan), East Asia (China, Indonesia, the Philippines), the Middle East (Saudi Arabia, Qatar, Egypt), and several European countries (Russia, Spain, Italy, Turkey, Romania, Ukraine). The second group of countries experienced smaller increases in average RSV levels compared to pre–COVID-19 levels (between 1 to 2.5 times; n=16), and largely comprised the United Kingdom, Germany, France, Netherlands, Portugal, Poland, Sweden, Belgium, Switzerland, Singapore, and Israel (Table S2, Multimedia Appendix 1). For most of these 40 countries, increases in RSV levels began in early March, either alongside or preceeding the rise in local COVID-19 case numbers, with the exception of China where the increase in Baidu RSVs was observed earlier during late January.

**Figure 4:**
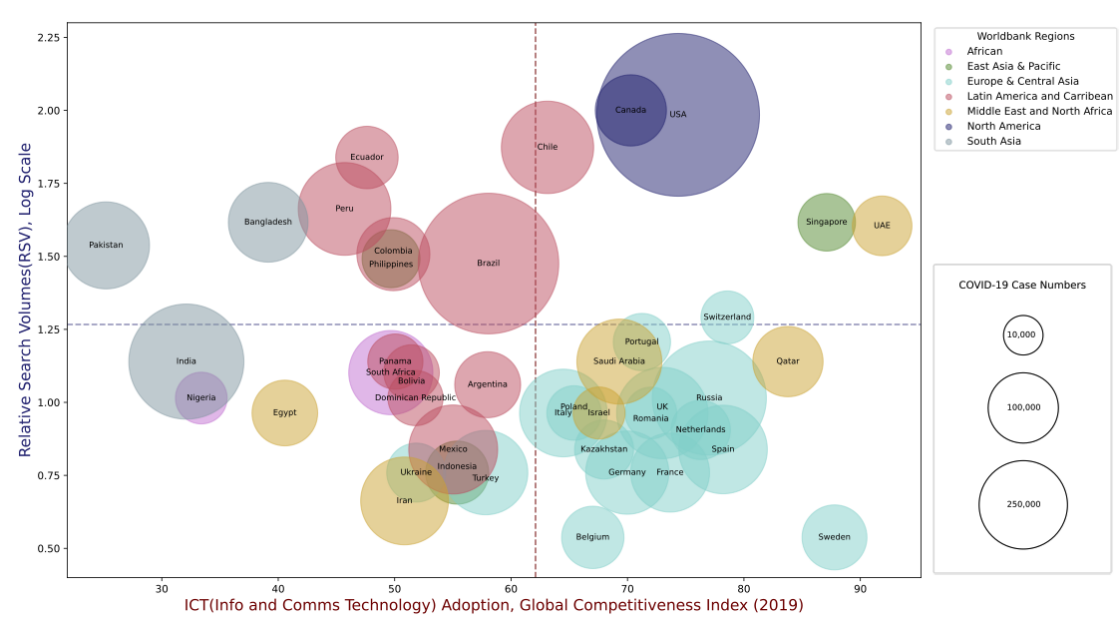
Relationship between telehealth-related relative search volumes (RSVs) versus information and communications technology (ICT) adoption index across the 50 countries most affected by COVID-19. Each country is represented as a data point and color-coded according to its World Bank region. The size of each plot reflects the accumulated total COVID-19 case numbers (as of July 7, 2020). The x axis represents the ICT adoption index, while the y axis represents the scaled (log) RSVs for each country. Vertical and horizontal dashed lines represent the mean values for the x and y axes, respectively. China and countries where RSV=0 (Google Trends) were not included in the plot.

Figure 4 illustrates the relationship between telehealth total-RSVs and ICT index values across the evaluated countries. There was no significant correlation between RSVs and ICT scores (*r*=0.11, *P*=.45; ρ=–0.08, *P*=.59). However, broad clustering patterns among countries in similar regions can be observed. By using the mean RSV value (horizontal dashed line) and mean ICT index (vertical dashed line), we visually divided the plot into 4 quadrants and observed that the United States and Canada occupy the top right quadrant, with high total-RSVs (≥96.6) and ICTs (≥70.3). The United Arab Emirates and Singapore were also in the top right quadrant, with similarly high total-RSVs (40.2 and 41.4, respectively) and ICT scores (91.9 and 87.1, respectively). In contrast, while European countries generally had high ICT scores (range 51.9-87.8), they had lower total-RSVs (range 3.4-19.5). Latin American countries generally occupied a cluster near the middle of the bubble plot, with moderate levels of total-RSVs (6.9-74.7) and moderate ICT scores (45.7-63.1). On the other hand, South Asian countries generally had moderate to moderate-high total-RSVs (13.8-41.4), despite their low ICT scores (≤39.1). Similarly, Figures S2 and S3 in Multimedia Appendix 1 illustrate the relationship between telehealth-RSVs with (1) the relative literacy rate and (2) the GDP per capita across the 50 countries. Similar clustering patterns of countries (as in the ICT evaluation) were observed.

## Discussion

### Principal Findings

In this study, we used data from GT, the Baidu Index, and Yandex Keyword Statistics to evaluate trends in telehealth demand during the first 6 months of the COVID-19 pandemic. To our knowledge, this is the first study to apply an infodemiological approach to investigate the potential public demand for telehealth in the 50 countries most affected by COVID-19. Our study further unraveled trends of demands alongside key COVID-19 events and varying levels of ICT infrastructure. Our findings suggest a general trend of increased demand for telehealth services across the evaluated countries during the COVID-19 pandemic. This trend was largely sustained beyond the initial wave, country lockdown periods, and subsequent reopenings. Our results suggest an ongoing and possible increased interest in telehealth services in the future as we enter a post–COVID-19 new normal phase. We also observed that current ICT infrastructure in several developing countries may lag behind this surging demand for telehealth. Our findings collectively indicate a pressing need to scale up telehealth capabalities in response to this growth in telehealth demand.

Our results demonstrate increased RSVs in most of the 50 evaluated countries. Among them, Canada and the United States had the highest total-RSVs and displayed a large spike in interest for telehealth services compared to most other countries. It is noteworthy that this pattern was also found in Australia (though not ranked within the top 50 countries), which had similarly high total-RSV values (RSV=98.9; data not shown in tables). Most other countries had less well-defined but observable increases in RSVs.

Interestingly, in many countries, RSV trends over time did not closely follow local COVID-19 case and death counts. However, RSVs often increased in early March, suggesting that the global announcement by the WHO on March 11, 2020, possibly had a great impact on the public’s change in behavior to seek for telehealth options. Other studies investigating COVID-19–related search trends have reported that increases in RSVs often preceeded local COVID-19 cases and deaths [17,33]. It has been suggested that RSV trends tend to change in response to particular “index events” [18]. For instance, Rovetta et al [33] reported that significant increases in COVID-19–related RSVs were only observed after the WHO’s COVID-19 pandemic announcement and the imposition of strict social distancing rules by governments.

Our study also found fair correlations between total-RSVs and a country’s COVID-19 cases and deaths. We posit two possible explanations for this. First, larger numbers of COVID-19 cases and deaths could result in increased risk perception in the general population, thus driving greater interest in telehealth to minimize risk exposures. This is in line with other studies that have also reported that changes in search volumes toward other risk-minimizing activities, such as social distancing, hand washing, and face mask wearing, corresponded with increases in COVID-19 cases [19,33]. Another potential explanation would be that the increase in telehealth-related RSVs reflected actual needs of affected patients. The timing of the RSV spikes for each country often preceded the actual spike in COVID-19 cases, suggesting that changes in search activity were probably more likely due to changes in public perception as the pandemic situation evolved. Nevertheless, further real-world investigations in the form of custom-designed questionnaires would be needed to further elucidate this aspect.

The bubble plots presented in this report further investigated the relationships between total-RSVs and various telehealth-related indices (ICT index, GDP per capita, literacy rates). These indices were evaluated as proxies for the capacity of a country to adopt and operationalize new telehealth services [34-36]. The clustering patterns observed in Figure 4 and Figures S2-S4 in Multimedia Appendix 1 may enable the classification of regions or countries based on their relative demands and capacity for telehealth services. For example, regions with the potential for rapid growth and adoption of telehealth are those with higher literacy levels, better ICT infrastructure, and higher RSVs (Figure S4, Multimedia Appendix 1). These countries include Argentina, Chile, Qatar, the United Arab Emirates, Saudi Arabia, and Singapore [37]. On the other hand, countries such as Colombia, Peru, Ecuador, Bangladesh, Pakistan, and the Philippines demonstrated growing interest in telehealth (moderate to high RSVs) but are limited by existing ICT capability (low to moderate ICT index).

Despite high ICT indexes and strong existing telehealth capacity (high CB/CBI scores; Figure S4, Multimedia Appendix 1) in a majority of European countries [38], the RSVs of these countries were relatively low compared to other developed western countries such as Canada and the United States. This observation may be explained by the availability and easy accessibility of existing telehealth services, which may also impact the population’s information-seeking behavior. For instance, in European countries that are at a more advanced stage of telehealth adoption, telehealth awareness and literacy may already be present, and patients may be directly seeking telehealth services from providers instead of conducting online queries in search engines [38]. Other factors that may also influence search volumes include different models of health care systems. For example, patients in the United Kingdom may contact their general practitioner or seek online consultations readily and directly through the NHS App [39]. Public health communications play a role as well. Sweden, for instance, contrarily downplayed the significance of the pandemic [40], eschewing lockdown measures, which may also explain why Sweden had the lowest RSVs among the evaluated European countries [40,41].

Our study offers useful insights into the short- and medium-term trends in telehealth demand in response to the COVID-19 pandemic. The trend of sustained increase observed in the Baidu Index RSV in China provides preliminary indications that telehealth demand will likely remain higher than in the pre–COVID-19 era. This trend is especially likely given that China can be considered to have entered its post–COVID-19 recovery phase, with minimal new cases reported over the last 3 months of our study period. On the other hand, given the resurgence of second or third waves of COVID-19 in several countries in recent months [1,42], it is foreseeable that telehealth demand may surge yet again or remain higher than the pre–COVID-19 period. Nonetheless, to better ascertain the long-term impact of COVID-19 on telehealth interest, further evaluation over a longer period is required.

### Strengths and Limitations

The key strengths of our study include our unique infoveillance approach to evaluate the public demand for telehealth services, capturing real-time responses to key COVID-19 pandemic events [11,16,43]. Second, our study provided extensive coverage of the 50 most affected countries worldwide, and evaluated these countries over a long duration (6 months), thus providing more concrete insights on trends. Third, we also included the additional use of the Baidu Index and Yandex Keyword Statistics to further investigate RSVs in China and Russia. The high degree of correlation between GT and Yandex Keyword Statistics for RSVs in Russia (*r*=0.875, *P*<.001) further confers a degree of reliability to our results.

This study also has a few limitations. First, it should be acknowledged that infodemiological approaches can only serve as a proxy for estimating the true demand for telehealth services. Second, as suggested earlier, although search engines provide a good catchment of overall interest, there may be alternative channels for the public to seek health care–related information, including their general practitioners, insurance services, or directly from telehealth service providers. In addition, it should also be acknowledgd that it is not possible to include all terms utilized by internet users to search for information on telehealth. Third, not all countries use Google as their primary search engine. Hence, using GT as a main proxy for overall demand when comparing across countries may be subjected to bias for some countries. Fourth, overall RSV trends may be confounded by different sampling profiles between countries, wherein factors including education and internet access may skew the representativeness of the RSV samples for each country. Additionally, infodemiology platforms such as GT and the Baidu Index present data as normalized RSVs rather than as absolute search counts, thus limiting direct comparisons of RSV data extracted from different sources of search engines [10]. Fifth, to reduce subjectivity, across all included countries (except for China), we standardized the definition of the post–COVID-19 period based on the date when the WHO declared COVID-19 as a global pandemic. Although it might have been ideal to individually quantify each country’s pre– and post–COVID-19 periods, it was difficult to do so, considering each country had a different community transmission trajectory. Furthermore, changes in search volumes were often in response to not just local events (lockdowns, local increases in COVID-19 cases, etc) but also global events such as the WHO’s pandemic announcement. Lastly, to better ascertain the long-term impact of COVID-19 on telehealth interest, further evaluation over a longer period is required.

### Conclusion

Telehealth is a major health technology solution that has gained further traction during the COVID-19 pandemic. We identified increased demand for telehealth services across the 50 countries most affected by COVID-19. We also found indications that several developing countries may still have suboptimal ICT infrastructure to cope with this surge in telehealth demand. These findings underscore a pressing need for policy makers and health care providers to scale up telehealth infrastructure and capability, during and beyond COVID-19.

## Appendix

Multimedia Appendix 1 Supplementary Information.

Multimedia Appendix 2 Time-trends for Telehealth-RSVs against daily COVID-19 cases in the top 50 countries most-affected by COVID-19. Countries arranged from left to right in order of total number of reported COVID-19 cases. x-axis represents time in individual days from January 1 to July 7, 2020. Left and right y-axes represent GT-RSVs and COVID-19 case numbers respectively. Red trendlines represent telehealth-related RSVs as measured by Google trends. Vertical bars in teal represent daily COVID-19 cases. Black vertical lines represent 2 key dates: the start of the Hubei Province lockdown (January 23, 2020) and the declaration of COVID-19 as a Pandemic by the World Health Organization (March 11, 2020). Shaded yellow regions represent country-specific lockdown or restriction periods. *Blue trendlines for China and Russia represent telehealth-related RSVs as measured by Baidu and Yandex respectively.

## Abbreviations

CB: Crunchbase
CBI: CB Insights
GDP: gross domestic product
GT: Google Trends
ICT: information and communications technology
RSV: relative search volume
WHO: World Health Organization
