# Supplementary material for: Telehealth Demand Trends During the COVID-19 Pandemic in the Top 50 Most Affected Countries: Infodemiological Evaluation
Source: JMIR Public Health Surveill. 2021 Feb 19;7(2):e24445. doi: 10.2196/24445 (PMC7899203; doi:10.2196/24445)
Supplement: Multimedia Appendix 1 [file publichealth_v7i2e24445_app1.pdf]

Supplementary Table 1: Search Strategy for Google Trends

Country	Search Combination	Languages	Official / Common Languages
**USA**	Telehealth + Telemedicine + Telemedizin + Télémédecine + Telesalud + Телемедицина + Telemedicina	English + German + Spanish + Russian + French + Italian	English
**Brazil**	Telehealth + Telemedicine + Telemedizin + Télémédecine + Telesalud + Telessaúde + Telemedicina	English + German + Portugese + Spanish + French + italian	Portugese
**India**	Telehealth + Telemedicine + टेलिमेडिसिन + Télémédecine + Telesalud + টেলিমেডিসিন + Telemedicina	English + Spanish + French + Hindi + Bangla + Marathi	Hindi, Bangla, Marathi
**Russia**	Telehealth + Telemedicine + Telemedizin + Télémédecine + Telesalud + Телемедицина + Telemedicina	English + German + Spanish + Russian + French + Italian	Russian
**Peru**	Telehealth + Telemedicine + Telemedizin + Télémédecine + Telesalud + Telessaúde + Telemedicina	English + German + Portugese + Spanish + French + italian	Spanish
**Chile**	Telehealth + Telemedicine + Telemedizin + Télémédecine + Telesalud + Telessaúde + Telemedicina	English + German + Portugese + Spanish + French + italian	Spanish
**UK**	Telehealth + Telemedicine + telefeddygaeth + teleiechyd + telezdrowie + telemedycyna + Telemedicina	English + Spanish + Welsh + Polish	English, Welsh, Polish
**Mexico**	Telehealth + Telemedicine + Telemedizin + Télémédecine + Telesalud + Telessaúde + Telemedicina	English + German + Portugese + Spanish + French + italian	Spanish
**Spain**	Telehealth + Telemedicine + Telemedizin + Télémédecine + Telesalud + Telessaúde + Telemedicina	English + German + Portugese + Spanish + French + italian	Spanish
**Iran**	Telehealth + Telemedicine + پزشکی از راه دور + teletıp + telesağlık	English + Turkish + Persian	Persian, Turkish
**Italy**	Telehealth + Telemedicine + Telemedizin + Télémédecine + Telesalud + Telessaúde + Telemedicina	English + German + Portugese + Spanish + French + italian	Italian, English, French, Spanish
**Pakistan**	Telehealth + Telemedicine + ٹیلی ہیلتھ + ٹیلی میڈیسن + ਟੈਲੀਮੀਡੀਸਾਈਨ + ਟੈਲੀਹੈਲਥ	English + Urdu + Punjabi	Urdu, English, Punjabi
**Saudi Arabia**	Telehealth + Telemedicine + الصحة عن بعد + التطبيب عن بعد	English + Arabic	English, Arabic
**Turkey**	Telehealth + Telemedicine + telemedizin + teletıp + telesağlık	English + German + Turkish	Turkish, English, German

**South Africa**	telemedisyne + Telehealth + Telemedicine + Telemedizin + Télémédecine + Telesalud + Telemedicina	English + German + Spanish + Russian + French + Afrikaans + Zuku + Xhosa	English, Zulu, Xhosa, Afrikaans
**Germany**	Telehealth + Telemedicine + Telemedizin + Télémédecine + Telesalud + Телемедицина + Telemedicina	English + German + Spanish + Russian + French + Italian	German
**Bangladesh**	Telehealth + Telemedicine + Telemedizin + Télémédecine + Telesalud + টেলিমেডিসিন + Telemedicina	English + German + Spanish + French + Italian + Bangla	Bangla / Bengali, English
**France**	Telehealth + Telemedicine + Télésanté + Télémédecine + Telesalud + telemedizin + Telemedicina	English + German + Portugese + Spanish + French + italian	French, English, Portugese, Italian, German
**Colombia**	Telehealth + Telemedicine + Telemedizin + Télémédecine + Telesalud + Telessaúde + Telemedicina	English + German + Portugese + Spanish + French + italian	Spanish
**Canada**	Telehealth + Telemedicine + Télésanté + Télémédecine + Telesalud + telemedizin + Telemedicina	English + German + Portugese + Spanish + French + italian	English, French
**Qatar**	Telehealth + Telemedicine + Telemedizin + Telesalud + Telemedicina + التطبيب عن بعد + الصحة عن بعد	English + German + Spanish + Arabic	Arabic, English
**China**	Telehealth + Telemedicine + 遠程醫療 + 远程医疗	English + Simplified and Traditional Chinese	
**Argentina**	Telehealth + Telemedicine + Telemedizin + Télémédecine + Telesalud + Телемедицина + Telemedicina	English + German + Spanish + Russian + French + Italian	Spanish
**Egypt**	Telehealth + Telemedicine + Telemedizin + Telesalud + Telemedicina + التطبيب عن بعد + الصحة عن بعد	English + German + Spanish + Arabic	
**Sweden**	Telehealth (topic)		English, Swedish, Finnish
**Indonesia**	Telehealth + Telemedicine + Telemedizin + Télémédecine + Telesalud + Телемедицина + Telemedicina	English + German + Spanish + Russian + French + Italian	Indonesian
**Belarus**	Telehealth (topic)		Belarusian, Russian
**Ecuador**	Telehealth + Telemedicine + Telemedizin + Télémédecine + Telesalud + Telessaúde + Telemedicina	English + German + Portugese + Spanish + French + italian	Spanish
**Iraq**	Telehealth + Telemedicine + Telemedizin + Telesalud + Telemedicina + التطبيب عن بعد + الصحة عن بعد		Arabic, Kurdish
**Belgium**	Telehealth + Telemedicine + Telegeneeskunde + Télémédecine + Телемедицина + telemedizin	English + Dutch + French + German + russian	Dutch, German, French

**UAE**	Telehealth + Telemedicine + телемедицина + Telesalud + Telemedicina + التطبيب عن بعد + الصحة عن بعد	English + Russian + Spanish + Arabic	Arabic, English
**Netherlands**	Telehealth + Telemedicine + Telegeneeskunde + Télémédecine + Телемедицина + telemedizin	English + Dutch + French + German + russian	Dutch
**Kuwait**	Telehealth + Telemedicine + телемедицина + Telesalud + Telemedicina + التطبيب عن بعد + الصحة عن بعد	English + Russian + Spanish + Arabic	Arabic, English
**Ukraine**	Telehealth + Telemedicine + Telemedizin + Télémédecine + Telesalud + Телемедицина + Telemedicina	Ukranian + Russian + English + German + French + Spanish	Ukranian, Russian
**Kazakhstan**	Telehealth + Telemedicine + Telemedizin + Télémédecine + Telesalud + Телемедицина + Telemedicina	Kazakh + Russian + English + German + French + Spanish	Kazakh, Russian
**Oman**	Telehealth + Telemedicine + телемедицина + Telesalud + Telemedicina + التطبيب عن بعد + الصحة عن بعد	English + Russian + Spanish + Arabic	Arabic
**Philippines**	Telehealth + Telemedicine + Telemedizin + Télémédecine + Telesalud + Телемедицина + Telemedicina	Filipino + English + German + Spanish + Russian + French + Italian	English and Filipino Official, Spanish common
**Singapore**	Telemedicine + Telehealth + 远程医疗	English + Chinese + Malay + Hindi	English Lingua Franca, Malay, Hindi and Chinese common, Malay official language
**Portugal**	Telehealth + Telemedicine + Telessaúde + Télémédecine + Telesalud + Télésanté + Telemedicina	Portugese + English + French + Spanish	Portugese, English, French and Spanish Common
**Panama**	Telehealth + Telemedicine + Telemedizin + Télémédecine + Telesalud + Telessaúde + Telemedicina	English + German + Portugese + Spanish + French + italian	Spanish
**Bolivia**	Telehealth + Telemedicine + Telemedizin + Télémédecine + Telesalud + Telessaúde + Telemedicina	English + German + Portugese + Spanish + French + italian	Spanish
**Dominican Republic**	Telehealth + Telemedicine + Telemedizin + Télémédecine + Telesalud + Telessaúde + Telemedicina	English + German + Portugese + Spanish + French + italian	Spanish
**Poland**	Telehealth + Telemedicine + Telemedizin + telemedycyna + telezdrowie + Телемедицина + Telemedicina	Polish + English + Russian + German + Spanish	Polish Official, English, Russian and German Common
**Afghanistan**	Telehealth + Telemedicine + تلیمیدیسین + پزشکی از راه دور	Pashto + Persian + English	Dari and Pashto & Persian
**Switzerland**	Telehealth + Telemedicine + Telemedizin + Télémédecine + Telesalud + Telessaúde + Telemedicina	English + German + Spanish + Russian + French + Italian	German, French, Italian and Romansh

**Israel**	Telehealth + Telemedicine + טלרפואה + Telesalud + Telemedicina + التطبيب عن بعد + الصحة عن بعد	English + Hebrew + Arabic + Spanish	Hebrew + Arabic
**Bahrain**	Telehealth + Telemedicine + телемедицина + Telesalud + Telemedicina + التطبيب عن بعد + الصحة عن بعد	English + Russian + Spanish + Arabic	Arabic Official, English Widely Spoken
**Nigeria**	Telehealth (Topic)		Official Language: English
**Romania**	Telehealth + Telemedicine + Telemedizin + Télémédecine + Telesalud + Телемедицина + Telemedicina	Romanian + English + German + Russian + Spanish	Romanian
**Armenia**	Telehealth + Telemedicine + հեռաբժշկություն + τηλεϊατρική + Telesalud + Телемедицина + Telemedicina	Armenian + English + Greek + Spanish + Russian	Armenian Official, Russian and Greek Popular

Supplementary Table 2: Additional RSV- and Telehealth-related parameters for top 50 countries most affected by COVID-19

	Country	Mean Pre-covid RSV levels	Mean Post-covid RSV levels	Ratio (Increase)	GDP-Per-Capita ($USD)	Literacy rates
1	**USA**	5.44	33.04	6.08	65,280.7	99
2	**Brazil**	7.15	41.22	5.76	8,717.2	93
3	**India**	6.26	30.72	4.91	2,104.1	74
4	**Russia**	21.03	58.37	2.78	11,585	99
5	**Peru**	6.09	29.90	4.91	6,977.7	94
6	**Chile**	3.94	35.07	8.89	14,896.5	96
7	**UK**	9.92	20.90	2.11	42,300.3	99
8	**Mexico**	14.07	20.08	1.43	9,863.1	95
9	**Spain**	13.01	34.62	2.66	29,613.7	98
10	**Iran**	10.69	10.93	1.02	5,520.3	86
11	**Italy**	11.33	39.74	3.51	5,995.1	99
12	**Pakistan**	0.84	15.86	18.82	1,284.7	59
13	**Saudi Arabia**	3.11	17.55	5.64	23,139.8	95
14	**Turkey**	1.60	14.42	9.01	9,042.5	96
15	**South Africa**	3.50	15.24	4.36	6,001.4	87
16	**Germany**	21.05	34.17	1.62	46,258.9	99
17	**Bangladesh**	2.20	21.53	9.80	1,855.7	74
18	**France**	13.33	24.44	1.83	40,493.9	99
19	**Colombia**	12.81	39.52	3.09	6,432.4	95
20	**Canada**	9.17	25.00	2.73	46,194.7	99
21	**Qatar**	4.07	11.42	2.81	64,781.7	93
22	**China**	18.6	58.1	3.13	10,261.7	97

23	**Argentina**	2.24	18.79	8.40	10,006.1	99
24	**Egypt**	5.02	12.96	2.58	3,020	71
25	**Sweden**	4.01	6.70	1.67	51,610.1	99
26	**Indonesia**	7.66	22.46	2.93	4,135.6	96
27	**Belarus**	0	2.23	NIL	6,663.3	99
28	**Ecuador**	6.04	18.93	3.13	6,183.8	93
29	**Iraq**	0	0	NIL	5,955.1	86
30	**Belgium**	2.77	6.63	2.39	46,116.7	99
31	**UAE**	11.35	35.21	3.10	43,103.3	93
32	**Netherlands**	8.27	18.69	2.26	52,447.8	99
33	**Kuwait**	0	0	NIL	32,032	96
34	**Ukraine**	7.82	22.46	2.87	3,659	99
35	**Kazakhstan**	2.78	5.52	1.98	9,731.1	99
36	**Oman**	0	0	NIL	15,474	96
37	**Philippines**	9.71	37.63	3.87	3,485.1	98
38	**Singapore**	13.22	26.54	2.01	65,233.3	97
39	**Portugal**	11.14	14.45	1.30	23,145	96
40	**Panama***	2.42	6.20	2.56*	15,731	95
41	**Bolivia***	3.24	8.78	2.71*	3,552.1	92
42	**Dominican Republic**	1.30	11.51	8.85	8,282.1	94
43	**Poland**	14.63	36.35	2.48	15,595	99
44	**Afghanistan**	0	0	NIL	502.1	43
45	**Switzerland**	15.85	17.65	1.11	81,993.7	99
46	**Israel**	5.88	13.89	2.36	43,641.4	92

47	**Bahrain**	0	0	NIL	23,504	97
48	**Nigeria**	7.91	18.60	2.35	2,229.9	62
49	**Armenia**	3.47	15.03	4.33	46,22.7	99
50	**Romania**	0	0	NIL	12,919.5	99
**Mean (SD):**				4.07±3.23	21,063 ± 21,011	92.22 ± 11.6

*Despite have RSV increases of (ratios) > 2.5 times, Panama and Bolivia were still considered to have experienced “smaller, subtler” changes in RSVs, due to the low RSV levels pre- and post-COVID (<10).

**Belarus was also considered to have “no observable trends” in RSVs due to pre-COVID RSV levels of 0.

Supplementary Figure 1: Worldwide Time-Trends for Telehealth-RSVs (blue) against daily COVID-19 deaths in top 50 countries

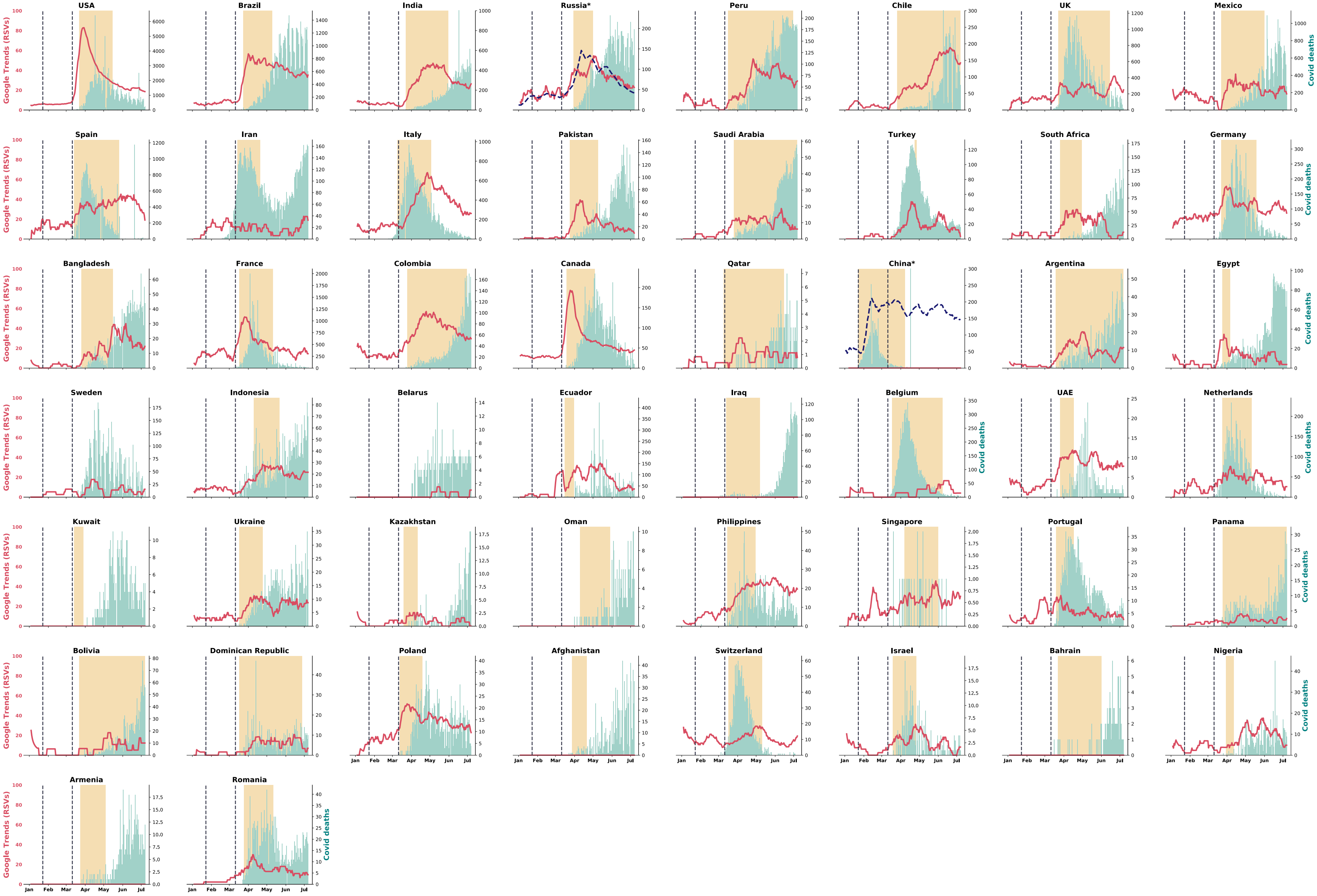

Countries arranged from left to right in order of total number of reported COVID-19 cases. x-axis represents time in individual days from 01/01/20 – 07/07/20. Left and right y-axes represent GT-RSVs and COVID-19 case numbers respectively. Red trendlines represent telehealth-related RSVs as measured by Google trends. Vertical bars in teal represent daily COVID-19 cases. Black vertical lines represent 2 key dates: the start of the Hubei Province lockdown (23rd January, 2020) and the declaration of COVID-19 as a Pandemic by the WHO (11th March 2020). Shaded yellow regions represent country-specific lockdown or restriction periods.

**Blue trendlines for China and Russia represent telehealth-related RSVs as measured by Baidu and Yandex respectivel*

Supplementary Figure 2: Relationship between Telehealth RSVs vs Literacy rates across the 50 most COVID-19 affected countries*.

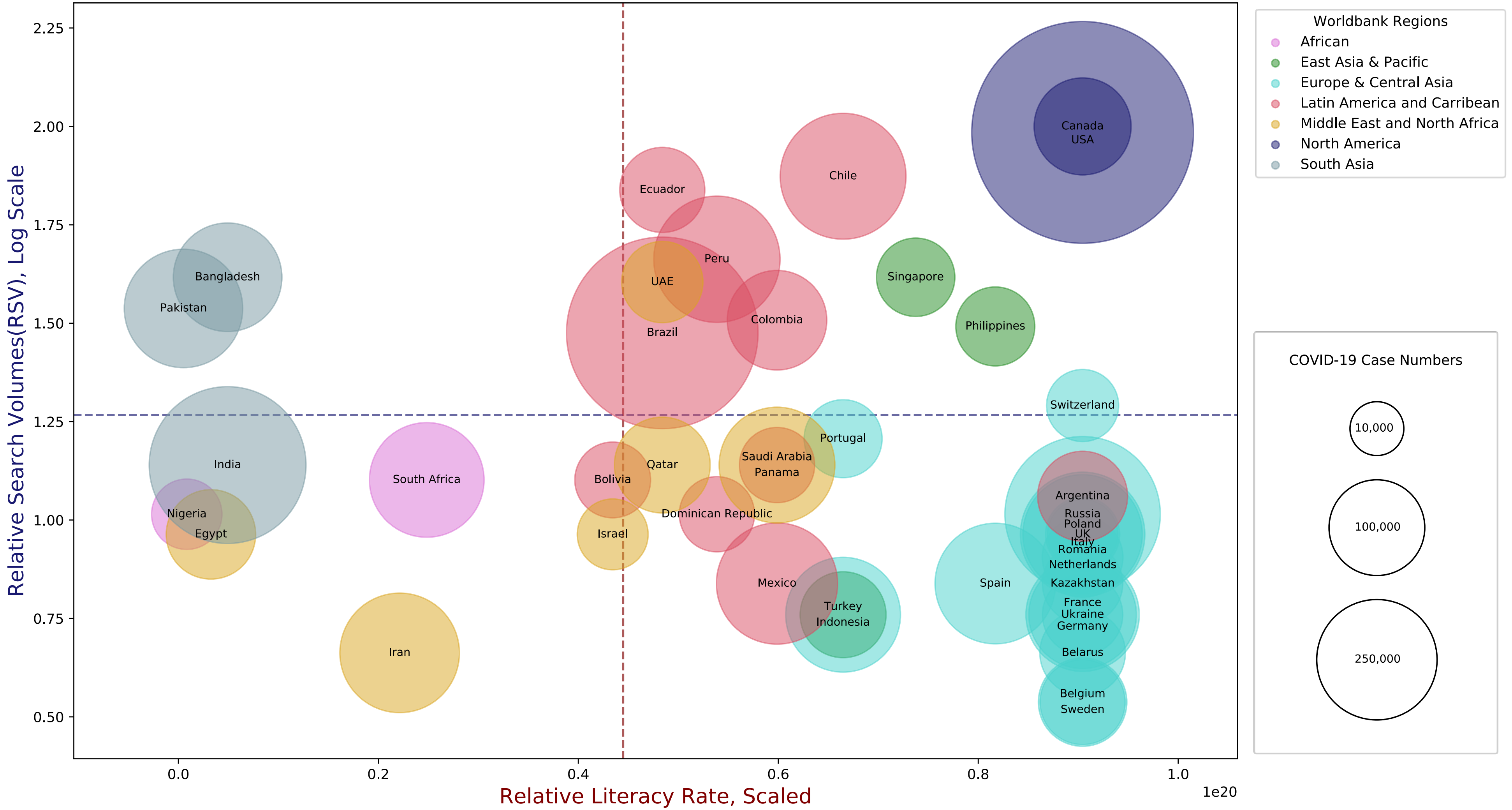

Each country is represented as a data-point, and color coded according to world-bank region. The size of each plot reflects the accumulated total COVID-19 case numbers (as of 7th July 2020). x-axis represents the ICT Development Index (ID), while the y-axis represents the scaled literacy rate for each country. Vertical and horizontal dashed lines represent the mean values for the x- and y-axes respectively.

**Countries with RSV=0 were not included in the plot.*

Supplementary Figure 3: Relationship between Telehealth RSVs vs GDP-per-capita ($USD) across the 50 most COVID-19 affected countries*.

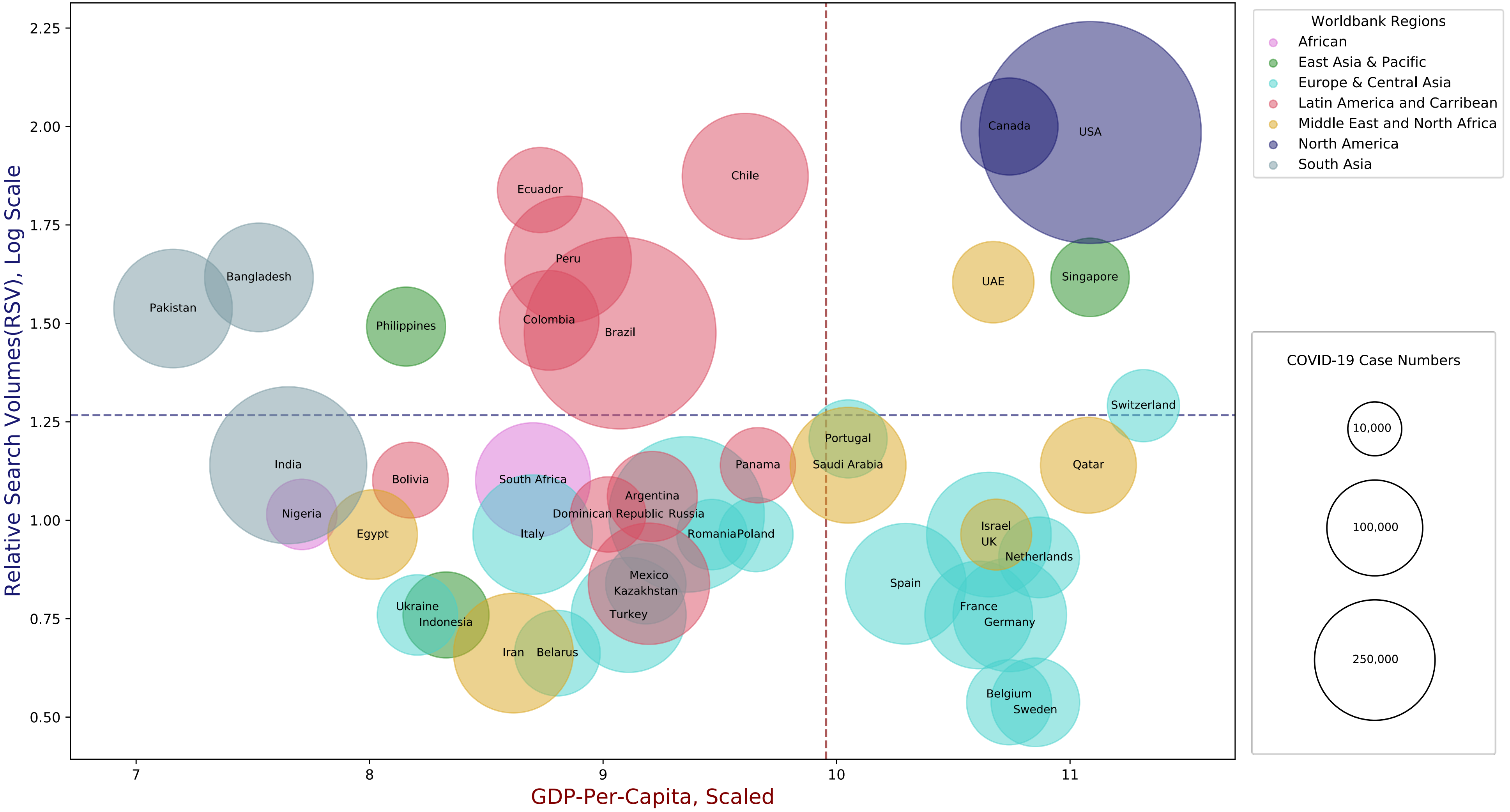

Each country is represented as a data-point, and color coded according to world-bank region. The size of each plot reflects the accumulated total COVID-19 case numbers (as of 7th July 2020). x-axis represents the ICT Development Index (ID), while the y-axis represents the (log) scaled GDP-per-capita for each country. Vertical and horizontal dashed lines represent the mean values for the x- and y-axes respectively.

**Countries with RSV=0 were not included in the plot.*

Supplementary Figure 4: Relationship between Telehealth RSVs vs ICT Adoption index across the 50 most COVID-19 affected countries*.

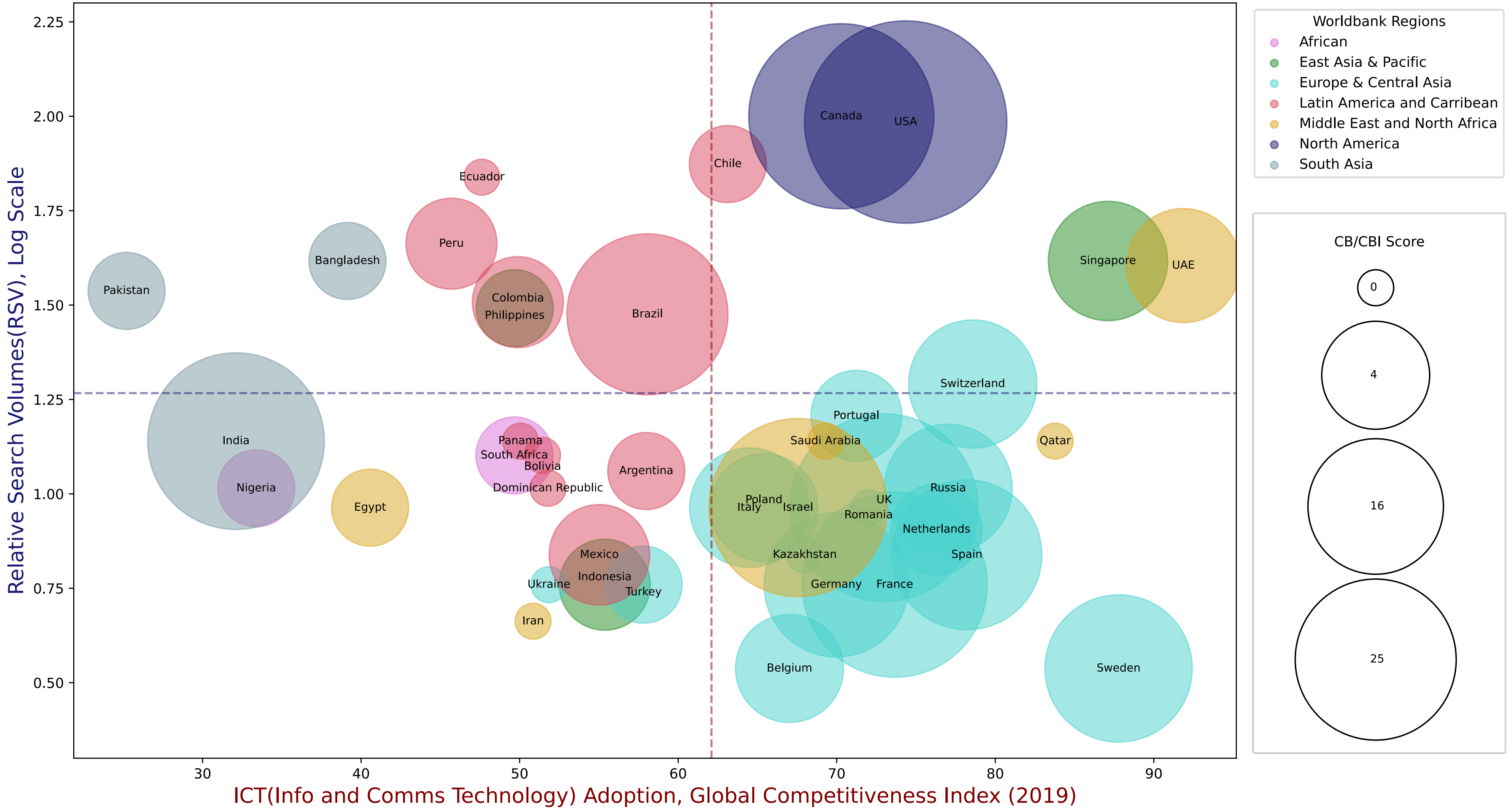

Each country is represented as a data-point, and color coded according to world-bank region. The size of each plot reflects the CB/CBI score (as a proxy for existing telehealth capacity). x-axis represents the ICT Adoption index, while the y-axis represents the scaled (log) RSVs for each country. Vertical and horizontal dashed lines represent the mean values for the x- and y-axes respectively.
**Countries with RSV=0 were not included in the plot.*
