# Supplementary figures and images for: Telehealth Demand Trends During the COVID-19 Pandemic in the Top 50 Most Affected Countries: Infodemiological Evaluation
Source: JMIR Public Health Surveill. 2021 Feb 19;7(2):e24445. doi: 10.2196/24445 (PMC7899203; doi:10.2196/24445)

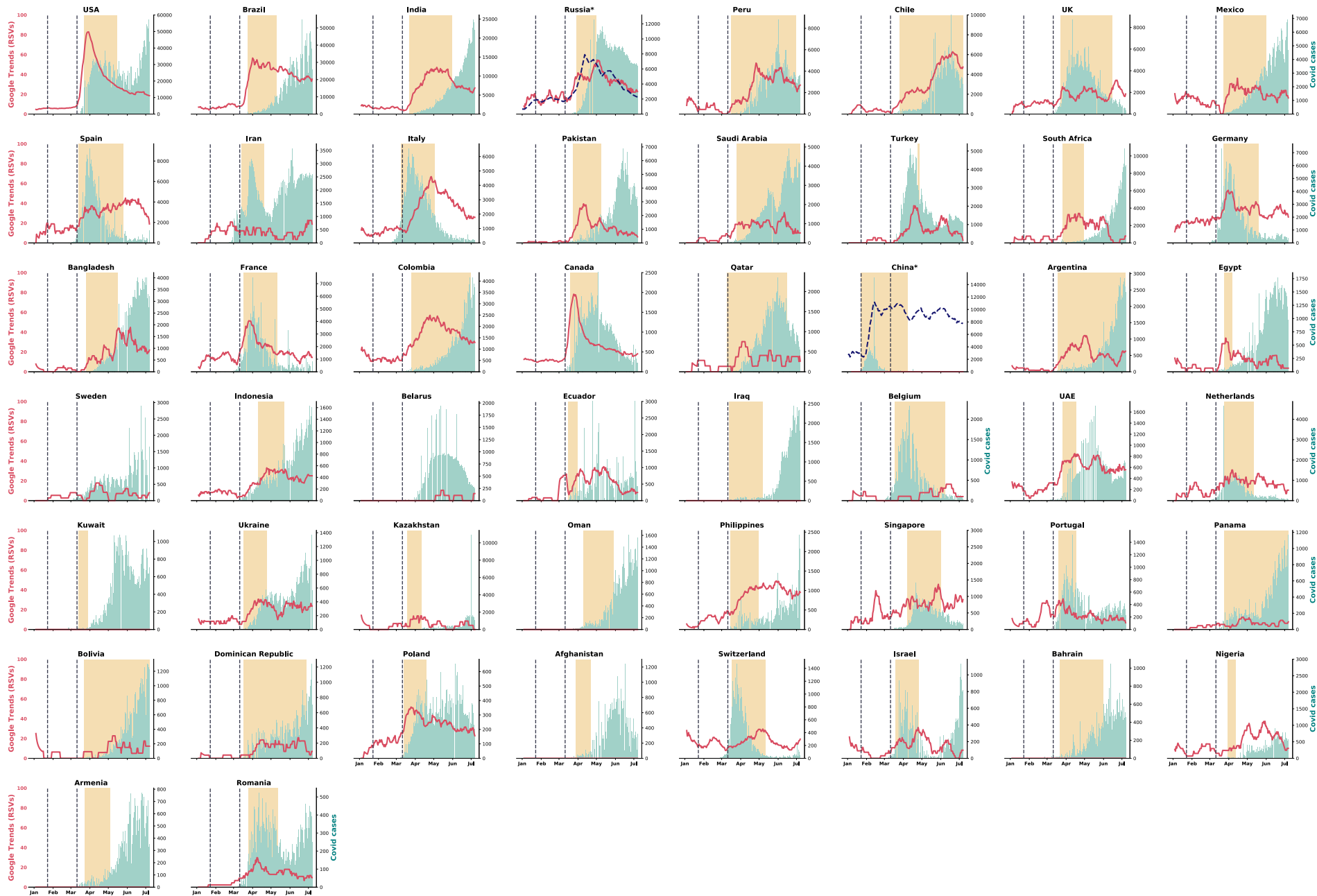

Supplement: Multimedia Appendix 2 [file publichealth_v7i2e24445_app2.pdf]
